# *Metallothionien 3 *expression is frequently down-regulated in oesophageal squamous cell carcinoma by DNA methylation

**DOI:** 10.1186/1476-4598-4-42

**Published:** 2005-12-13

**Authors:** Eric Smith, Paul A Drew, Zi-Qing Tian, Neville J De Young, Jun-Feng Liu, George C Mayne, Andrew R Ruszkiewicz, David I Watson, Glyn G Jamieson

**Affiliations:** 1 Department of Surgery, The University of Adelaide, Royal Adelaide Hospital, Adelaide, South Australia, 5005, Australia; 2 School of Nursing and Midwifery, Flinders University, Bedford Park, South Australia, 5042, Australia; 3 Department of Thoracic Surgery, Fourth Hospital of Hebei Medical University, Shijiazhuang, Hebei, P.R. China; 4 Department of Surgery, Flinders University, Bedford Park, South Australia, 5042, Australia; 5 Division of Tissue Pathology, Institute of Medical and Veterinary Science, Adelaide, South Australia, 5000, Australia

## Abstract

**Background:**

Metallothionein 3 (MT3) inhibits growth in a variety of cell types. We measured *MT3 *gene expression by RT-PCR, and DNA methylation in the *MT3 *promoter by combined bisulphite restriction analysis, in four oesophageal cancer cell lines and the resected oesophagus from 64 patients with oesophageal squamous cell carcinoma (SCC).

**Results:**

*MT3 *expression was not detected in one of the four oesophageal cell lines. The *MT3 *promoter was methylated in all of the oesophageal cell lines, but the degree of methylation was greater in the non-expressing cell line. After treatment with 5-aza-2'-deoxycytidine there was a reduction in the degree of methylation, and an increase in *MT3 *expression, in each of the cell lines (p < 0.01). Methylation was detected in 52% (33 of 64) of primary SCC and 3% (2 of 62) of histologically normal resection margins. *MT3 *expression was measured in 29 tumours, 17 of which had methylation of *MT3*. The expression of *MT3 *was significantly less in the methylated tumours compared to either the unmethylated tumours (p = 0.03), or the matched margin (p = 0.0005). There was not a significant difference in *MT3 *expression between the tumour and the margin from patients with unmethylated tumour. No correlations were observed between methylation of *MT3 *and survival time, patient age, gender, smoking or drinking history, tumour stage, volume, or lymph node involvement.

**Conclusion:**

We conclude that *MT3 ex*pression is frequently down-regulated in oesophageal SCC, by DNA methylation, but that this is not a prognostic indicator.

## Background

The metallothioneins (MT) are a group of low molecular weight, cysteine-rich intracellular proteins that are involved in maintaining intracellular metal homeostasis by binding transition metals such as zinc and copper. There are 10 functional isoforms of MTs described, which are divided into 4 classes, designated MT1 – 4, on the basis of small differences in protein sequence and charge characteristics [1,2]. The MTs have been proposed to play an important role in protecting against DNA damage, apoptosis and oxidative stress [3].

Metallothionein 3 (MT3) was first identified as a growth inhibitory factor, expressed in normal brain, which inhibited the survival of neurones in culture and also neurite formation [4]. Subsequent studies using glial [5] or tumour[6-8] cells, stably transfected with *MT3*, showed that its endogenous over-expression could inhibit cell growth. More recently, down-regulation of *MT3 *was reported as one of 17 changes in gene expression which was most likely to be associated with metastasis and poor clinical outcome in a range of solid tumours [9]. One mechanism for reducing gene expression is methylation of the CpG island when present in the promoter region of the gene [10]. Methylation of the *MT3 *promoter has been observed and has been suggested to cause reduced expression in gastric cancer [11].

The levels of *MT3 *expression, the frequency of *MT3 *methylation, and correlations between *MT3 *methylation and clinical parameters, have not been investigated in oesophageal squamous cell carcinoma (SCC). In this study we used combined bisulfite restriction analysis (COBRA) [12] to estimate the frequency of methylation at specific sites within the *MT3 *promoter in oesophageal cancer cell lines, and investigated the relationship between methylation and its expression by quantitative real-time RT-PCR. We then measured *MT3 *gene expression and the frequency of *MT3 *methylation in primary oesophageal SCCs and, when available, the histologically normal, proximal resection margin from those patients.

## Results

### Methylation analysis of *MT3 *in normal lymphocytes and oesophageal cell lines

**Figure 1:**
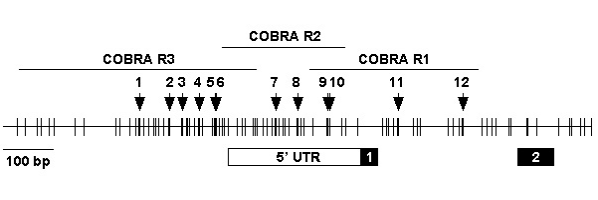
**Schematic diagram of the *MT3 *promoter**. The diagram was generated by downloading the *MT3 *CpG island genetic element with the addition of 200 bases upstream from , using the Human July 2003 Freeze. The location of 5' UTR (□), exon 1 and exon 2 (■) of *MT3 *(NM_005954) are shown. Each vertical line represents a CpG. The arrows represent the location of the 12 BstUI restriction enzyme sites analysed. The regions amplified for the 3 COBRA PCRs, R1, R2 and R3, are shown.

The methylation status of the *MT3 *CpG island was measured by COBRA within 3 overlapping regions (Figure 1). Complete digestion of the COBRA PCR product indicated methylation at one or more of the BstUI sites within that region of all alleles. Incomplete digestion indicated methylation at one or more of the BstUI sites within that region of some but not all alleles.

COBRA was performed on the bisulphite modified lymphocyte DNA from 19 different normal donors without any known cancers or other genetic abnormalities. In each of these donor lymphocytes samples an average of two percent (± 1% SD) of molecules were methylated at site 12 in the R1 region, located in intron 1. There was no evidence of methylation at any of the other sites analysed by COBRA in the R1 and the R2 regions (data not shown).

**Figure 2:**
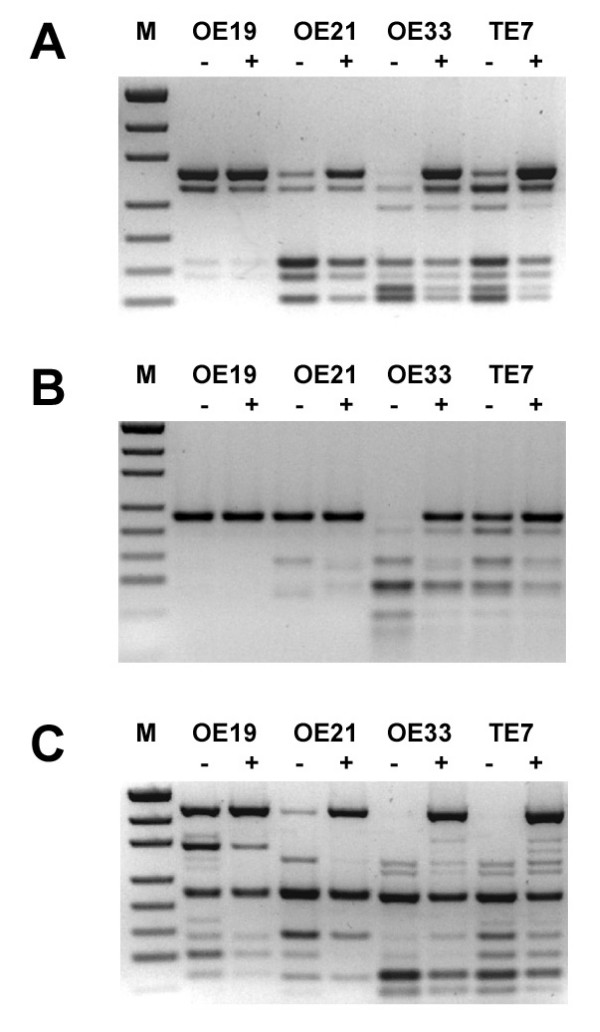
**The *MT3 *COBRA on regions 1 (A), 2 (B) and 3 (C) for the oesophageal cell lines with or without treatment with Aza-dC**. The COBRA was performed on the oesophageal cell lines following 72 to 96 hrs of treatment with either 1 μM aza-dC (+) or vehicle (-). M, molecular weight marker.

COBRA was performed on the oesophageal cancer cell lines OE19, OE21, OE33 and TE7, cultured with or without the DNA demethylation drug 5-aza-2'-deoxycytine (aza-dC) (Figure 2). Complete digestion of each of the 3 regions was observed for OE33. For TE7, incomplete digestion was observed in the R1 and R2 regions, whilst complete digestion was observed in R3 region. Incomplete digestion was observed in each of the 3 regions for OE21. For OE19, no digestion was observed in the R2 region, but incomplete digestion was observed in the R1 and R3 regions. The frequency of methylation at any BstUI site within each of the regions, R1, R2 or R3, was estimated in the oesophageal cell lines (Table 1). The frequency of methylation in each region varied from cell line to cell line, and the frequency of methylation in a given cell line varied from region to region. We then estimated the frequency of methylation at each BstUI site within a region (Table 2). We observed that the frequency of methylation at each BstUI site within a region varied for a given cell line and varied between cell lines.

**Table 1:** The frequency of methylation of any BstUI site within a region, in oesophageal cell lines. The estimated frequency of methylation of any BstUI site within R1, R2 or R3, in the oesophageal cancer cell lines treated without (-) or with (+) with 1 μM aza-dC, where Δ is the change in methylation frequency. The results are from a single, representative experiment.

	OE19			OE21			OE33			TE7		
	-	+	Δ	-	+	Δ	-	+	Δ	-	+	Δ

R1	32%	23%	-10%	93%	57%	-37%	97%	37%	-60%	88%	33%	-55%
R2	6%	6%	0%	15%	10%	-5%	96%	32%	-64%	51%	19%	-32%
R3	73%	30%	-43%	97%	40%	-57%	95%	51%	-45%	100%	37%	-63%

**Table 2:** The frequency of methylation of any BstUI site within R1, in oesophageal cell lines. The estimated frequency of methylation at each BstUI site of R1 in the oesophageal cancer cell lines treated without (-) or with (+) with 1 μM aza-dC, where Δ is the change in methylation frequency. The results are from a single, representative experiment.

	OE19			OE21			OE33			TE7		
	-	+	Δ	-	+	Δ	-	+	Δ	-	+	Δ

9/10	4%	5%	1%	34%	18%	-16%	60%	13%	-47%	36%	9%	-27%
11	7%	6%	-1%	88%	48%	-40%	85%	16%	-69%	55%	13%	-42%
12	30%	21%	-10%	76%	48%	-29%	91%	34%	-57%	82%	30%	-52%

### Re-expression of the *MT3 *after 5-aza-2'-deoxycytine treatment

**Figure 3:**
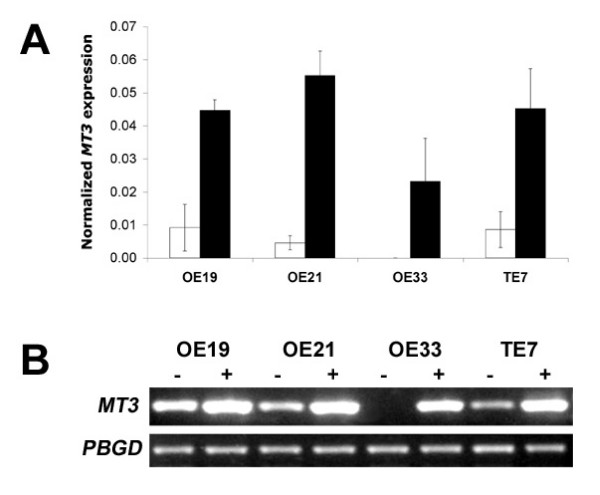
**The expression of *MT3 *in oesophageal cell lines**. A) *MT3 *expression in the oesophageal cell lines grown for 72 to 96 h with either 1 μM aza-dC (■) or vehicle (□). Results are the average of triplicate values ± SD, normalised to that of *PBGD*. B) Representative example of gel electrophoresis of the *MT3 *and *PBGD *RT-PCR products for the cell lines following treatment with either 1 μM aza-dC (+) or vehicle (-).

To determine the relationship between methylation and transcription of the *MT3 *gene, we measured the expression of *MT3 *by RT-PCR in 4 oesophageal cell lines, OE19, OE21, OE33 and TE7, cultured in the presence or absence of aza-dC (Figure 3). *MT3 *expression was undetectable in the completely methylated cell line OE33. Low levels of *MT3 *were measured in the partially methylated cell lines OE19, OE21 and TE7. Demethylation treatment with aza-dC reduced the frequency of methylation both within and between different regions for all cell lines provided that the intial frequency of methylation was ≥ 10 %. Demethylation treatment did not completely demethylate all the cells in culture. The expression of *MT3 *was significantly increased following aza-dC treatment in each of the oesophageal cell lines (p < 0.01). The increase in *MT3 *expression induced by aza-dC was significantly less in the OE33 cell line compared to the OE19, OE21 or TE7 (p = 0.02).

### Methylation and expression of *MT3 *in oesophageal SCC

The frequency of *MT3 *methylation was measured in resected primary tumour and, when available, the proximal resection margin from 64 patients with oesophageal SCC. From each sample the R1 and R2 regions of the *MT3 *CpG island were amplified by PCR and then analysed for methylation by COBRA. As with the lymphocyte samples, low levels of methylation at BstUI site 12 in the R1 region were observed in all the histologically normal margins and the tumour samples from these patients. Thus, this ubiquitous low level of methylation was not considered in the subsequent analysis. Methylation of the R1 region was observed in 33 (52%) and of the R2 region in 10 (16%) of the tumour samples. All samples that were methylated in the R2 region were also methylated in the R1 region. No correlations were observed between patient age, gender, smoking or drinking history, tumour, volume, or lymph node involvement and the methylation of *MT3 *(Table 3). Methylation of the R2 region was observed in none of the 62 proximal resection margin tissues available, whilst methylation of the R1 region was observed in 2 (3%).

**Table 3:** The incidence of *MT3 *methylation for the 64 patients with oesophageal SCC.

	*n*	Unmethylated	Methylated
Male	45	22	23
Female	19	9	10
Age, median (range)	57 (42 – 76)	57 (46 – 76)	58 (42 – 70)
Males	57 (42 – 70)	54 (46 – 68)	57 (42 – 70)
Females	62 (49 – 76)	63 (49 – 76)	61 (50 – 68)
Tumour volume (cm^3^), median (range)	57 (4 – 300)	54 (4 – 300)	60 (4 – 225)
Histological differentiation			
Well/moderate	57	28	29
Poor	4	2	2
Not recorded	3	3	0
Tumour stage			
T1N0M0	3	1	2
T2N0M0	9	5	4
T2N1M0	3	2	1
T2N2M0	1	1	0
T3N0M0	29	13	16
T3N1M0	9	6	3
T3N2M0	1	0	1
T3N2M1	1	0	1
T4N0M0	2	2	0
T4N0M1	1	0	1
T4N1M0	2	1	1
Not recorded	3	0	3

**Figure 4:**
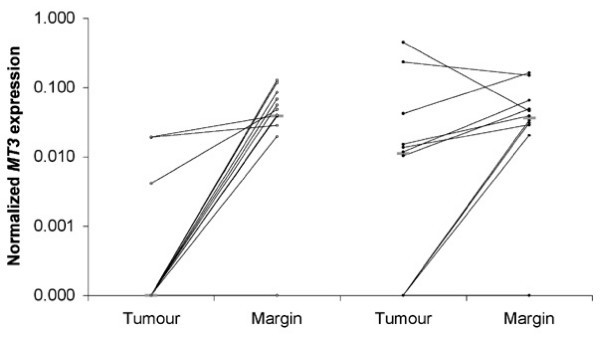
***MT3 *expression in patient-matched tumour and non-cancerous proximal resection margin from patients with SCC of the oesophagus**. *MT3 *expression in the oesophageal tissue from patients with either methylated tumour (○) or umethylated tumour (●). Results are the average of triplicate values, normalised to that of *ACTB*. The grey bars represent the median expression for each group. The black lines link expression values of tissues from the same patient.

*MT3 *gene expression was analysed by quantitative real-time RT-PCR in 29 of the primary tumours and their matched non-cancerous proximal resection margins for which RNA was available. *MT3 *was methylated in 17 of these tumours, and unmethylated in 12. The *MT3 *expression was normalized to that of *ACTB*. The expression of *MT3 *was significantly less in the tumours in which the promoter was methylated compared to those in which it was unmethylated (Figure 4, p = 0.03). *MT3 *expression in methylated tumours was significantly less than that of the matched margin (p = 0.0005). There was not a statistically significant difference in expression between the tumour and matched margin from patients in whom the tumour was unmethylated.

Survival data was available for 41 patients, 19 with an unmethylated tumour, 22 methylated. There was no difference between these 2 groups with respect to the time since operation (unmethylated: range 3 – 22 months, median 20 months; methylated: range 1 – 22 months, median 21 months). There were 5 deaths in the unmethylated and 4 in the methylated group (P = 0.7). There was no difference in the survival time following operation, with deaths in the unmethylated group occurring 3, 13, 13, 17 and 18 months post-operatively, compared to 1, 4, 8 and 22 months in the methylated group.

## Discussion

In this study we investigated the expression of *MT3 *and its control by DNA methylation in oesophageal SCC. We measured methylation of *MT3 *by COBRA in three separate regions of its promoter, and gene expression by quantitative real-time RT-PCR. In the oesophageal cell line OE33, which had complete methylation at all the CpG sites analysed, there was also complete transcriptional silencing of the *MT3 *gene. In a further 3 cell oesophageal cancer cell lines, each of which had partial methylation, there was a partial but not complete reduction in gene expression. Treatment of each of these cell lines with aza-dC, which reduced methylation, resulted in an increase in gene expression.

In those the cell lines which had partial methylation, the amount of methylation could vary between contiguous CpGs in the same cell line, and could vary in particular CpGs between cell lines. Thus, a particular CpG which might be unmethylated in one cell line could be partially or completely methylated in other cell lines expressing similar levels of *MT3*. This is consistent with the reported variability of methylation of the p16 CpG island in primary human mammary epithelial cells during escape from growth arrest [13], and the O6-methylguanine-DNA methyltransferase gene in human cell lines [14]. The significance of this finding is that methods for the measurement of DNA methylation such as methylation specific PCR (MSP) which analyse methylation at one or two CpGs only may misrepresent the methylation pattern of a region. In addition these methods would mislead if they probed CpGs with low levels of methylation in all tissues analysed, such as the site 12 in intron 1 in our analysis of *MT3*. This was methylated in normal lymphocytes and normal oesophageal tissue, as well oesophageal SCC, even though other CpGs were unmethylated and gene expression was high.

In order to show the relationship between methylation within the regions studied and MT3 gene expression, we incubated cultures of the oesophageal cell lines for approximately two cell divisions with aza-dC, a potent inhibitor of DNA methyltransferase. Because the rate of division varied for each of the cell lines, we determined the length of time required for each of the cell lines to divide twice in the presence of aza-dC using PKH-26 labelling [15]. This stable red fluorescent dye inserts into the cytoplasmic membrane, and is distributed equally amongst each of the daughter cells at the time of cell division, such that the mean fluorescence intensity of the cell population is halved with each cell division. As expected, treatment with aza-dC for two cell divisions did not completely demethylate the cell lines. This is because it is only newly synthesised strands which are demethylated, so if in a cell a particular region of DNA is completely methylated on both alleles, after 2 divisions only 75% of the alleles will be demethylated. We found that there was a significant reduction in the amount of methylation at each of the methylated sites analysed following drug treatment. Interestingly, the amount of the reduction varied between different CpG sites within a cell line, and varied from cell line to cell line at a given site. The reasons for this cell line and regional variation in the extent of demethylation are unknown.

Previous studies have shown that the methylation of only a subset of available CpG sites within a region can be sufficient to reduce transcription [16-18,13], with a greater reduction in transcription as the density of CpG methylation increases [19]. Complete methylation of all the sites in the regions which we studied correlated with lack of any gene expression, while methylation of only a subset of sites correlated with some *MT3 *expression, and the further reduction in methylation resulting from demethylation with aza-dC always correlated with an increase in *MT3 *expression. In this study we evaluated *MT3 *expression only by RT-PCR, because an antibody specific for the MT3 protein was not commercially available. However, previous studies have shown that when the *MT3 *transcript was able to be detected the protein was also detectable [20,21,7,8].

Our interest in *MT3 *came from a report that solid tumours with a specific pattern of expression of 17 genes, including down-regulation of *MT3*, were most likely to be associated with metastasis and poor outcome [9]. Oesophageal SCC is an aggressive disease with a 5 year survival of about 20%, death most commonly due to secondaries. Molecular markers which assist in predicting metasases might help to tailor treatment options better. *MT3 *was first identified as a growth inhibitory factor which decreased the survival of neuronal cultures [4]. Subsequent studies using stably transfected cell lines showed that up-regulation of *MT3 *was growth inhibitory in some, but not all, cell lines [6-8].

The reported role of *MT3 *in carcinogenesis is unclear. In gastric carcinomas *MT3 *expression was found to be markedly reduced, but there was no indication of any relationship to outcome in this report [22]. In contrast, levels of *MT3 *protein were shown to be elevated in bladder [20] and breast carcinomas [21]. This elevated expression was a poor prognostic indicator, being associated with increased tumour stage in bladder carcinoma, and poor disease outcome in some breast carcinomas. In our series of 64 patients with oesophageal SCC we found a degree of methylation in 52% of primary tumours and 3% of the histologically normal proximal resection margin. We observed that *MT3 *expression was frequently down-regulated in primary oesophageal SCC when compared to normal mucosa, and significant down-regulation was most commonly observed in those tumours that were methylated for *MT3*, and not unmethylated tumours. Interestingly, 3 tumours which had methylated DNA also had normal expression of *MT3*. This might reflect a heterozygous pattern of methylation, with only one allele methylated, or a mixed tumour cell population containing some cells which were methylated and some which were not. The techniques used in this study could not distinguish between these possibilities. Also, 3 patients with unmethylated DNA had low levels of *MT3 *expression, perhaps reflecting mutation or other change in the tumour. We found no relationship between methylation status and survival in the subset of patients for whom data were obtainable, suggesting that in these patients there would also be no relationship between gene expression and survival. Methylation and reduced expression of *MT3 *was also not associated with tumour stage or tumour size. Thus a change in *MT3 *expression by itself does not appear to favour increased tumour growth, and methylations status is not associated with survival, in patients with oesophageal SCC.

## Conclusion

We have shown that the *MT3 *promoter has a heterogeneous pattern of methylation in oeosphageal cancer cell lines, and is associated with a reduction in the gene expression. In resected oesophageal SCC tissue *MT3 *expression was frequently down-regulated, most commonly in those tumours in which the promoter region was methylated. Methylation or down-regulation of *MT3 *did not correlate with and patient age, gender, smoking or drinking history, tumour stage, volume, lymph node involvement, or patient survival. MT3 methylation does not show promise as a prognostic marker in patients with oesophageal SCC.

## Methods

### Cell lines and tissue samples

The oesophageal cancer cell lines OE19, OE21, OE33 and TE7 [23] were each cultured in RPMI 1640 supplemented with 10% foetal bovine serum at 37°C with 5% CO_2_. Whole blood was obtained from 19 normal donors, none of whom had known cancers or genetic abnormalities. Primary tumour and, when available, non-cancerous proximal resection margin from 64 consecutive patients undergoing oesophagectomy for SCC at the Department of Thoracic Surgery, Fourth Hospital, Hebei Medical University, were collected into RNAlater (Ambion, Austin, TX). Patient gender, age at the time of operation, smoking and drinking history and tumour type, stage, volume, and conventional histopathology were recorded. Survival data were available for 41 patients. The study complied with the appropriate institutional guidelines.

### Demethylation of oesophageal cell lines by 5-aza-2'-deoxycytine treatment

Re-expression studies for *MT3 *were performed on each of the oesophageal cancer cell lines. To determine the culture conditions required to achieve at least 2 cell divisions in the presence or absence of 5-aza-2'-deoxycytine (aza-dC), cells, labelled with PKH-26 (Sigma-Aldrich, Sydney, NSW, Australia), were analysed by flow cytometry as described previously [15]. The cells were treated for 72 (OE21, OE33 and TE7) or 96 (OE19) hr with culture medium containing either vehicle or 1 μM aza-dC (Sigma-Aldrich). The medium was then replaced with fresh medium not containing aza-dC, and the cells were incubated for a further 24 hr before harvesting.

### Preparation of bisulphite modified DNA

Genomic DNA was isolated from normal donor lymphocytes, cultured cells, and RNAlater stabilized tissues. Lymphocytes were isolated from whole blood using Ficoll-Paque (Pharmacia Biotech, Uppsala, Sweden). The adherent cell lines were harvested by detaching the cells from culture flasks using trypsin/EDTA. RNAlater stabilized tissues were homogenised using disposable pestles (Edwards Instruments, Narellan, NSW, Australia). Next, the samples of lymphocytes, cultured cells, or RNAlater stabilised tissues were digested for 3 days with proteinase K and sodium dodecyl sulfate in TES, pH 8. The protein was removed by NaCl precipitation. The DNA was precipitated with ethanol, and resuspended in TE, pH 8. The DNA was bisulphite modified using a modification of the method previously described [24]. Briefly, 2 μg of genomic DNA was denatured by treatment with NaOH at 37°C for 15 min. The DNA was modified using a mixture of 5 M sodium bisulfite (Sigma-Aldrich) and 0.72 μM hydroquinone (Sigma-Aldrich) for 4 hr at 56°C. The samples were purified using Rapid PCR Purification System (Marligen Biosciences, Ijamsville, MD), desulphonated with NaOH for 15 min at 37°C, precipitated with ethanol and sodium acetate, and resuspended in 50 μl of TE, pH 8.

### Methylation analysis of the *MT3 *promoter by COBRA

Bisulphite modified DNA was amplified using primers that amplified 3 overlapping regions, designated R1, R2 and R3 (Figure 1). The primers did not discriminate between methylated and unmethylated alleles. The primers and PCR conditions were specific for bisulphite modified DNA, and did not amplify unmodified DNA. All COBRA PCRs were performed in a volume of 50 μl containing 2 μl of bisulphite modified DNA, 2 mM MgCl_2_, 0.2 mM dNTPs, 0.2 μM of forward and reverse primer, and 0.5 U of HotStar Taq in 1 × PCR Buffer (Qiagen, Hilden, Germany). The reactions were incubated in an Eppendorf Mastercycler at 95°C for 15 min; then 45 cycles of 94°C for 1 min, 52, 54 or 60°C for 1 min, and 72°C for 1 min; and a 4 min final extension at 72°C (Table 4). The PCR products (5 μl) were digested with 5 units of BstUI restriction enzyme (New England Biolabs, Beverly, MA) in a final volume of 10 μl, for 4 hr at 60°C. BstUI specifically digests at CGCG sites that are retained after bisulphite modification when CpGs are methylated. The unmethylated cytosines are deaminated to uracil by the bisulphite reaction, then amplified as thymine in the PCR reaction, and so are not digested by BstUI. The digested PCR products were resolved on 2% agarose gels and stained with ethidium bromide. The intensity of each band was quantified and converted to nanograms of DNA using Kodak ID Image Analysis Software (Kodak, Rochester, NY). For each gel, 250 ng of the molecular weight marker pUC19/Hpa II (GeneWorks, Adelaide, SA, Australia) was used as a standard to determine DNA fragment size and mass. Restriction maps for each digest were used to determine the length of all possible fragments, and the number of molecules in each band were determined by multiplying the mass of DNA in each band by its' fragment length. The frequency of methylation at each BstUI site was then estimated by dividing the number of molecules for each fragment by the total number of molecules in the digest.

**Table 4:** Primer sequences and related information.

Primer	Genome position^a^	Primer sequence	Annealing temperature	Product size
R1-forward	+148	5'-AGTATYGTTATTTGTTGTTATTAGTTAAGT-3'	54°C	298 bp
R1-reverse	+445	5'-TAAAATACCAAATCTCCCTATTCTC-3'		
R2-forward	-8	5'-GAGYGGGTTTYGGTAGTGTATATAT-3'	52°C	217 bp
R2-reverse	+209	5'-TAAACRAACTTCTCCAAACAACTAAAC-3'		
R3-forward	+58	5'-GAAATGGAATAYGTTTTTTGTTAGTGAT-3'	60°C	427 bp
R3-reverse	-369	5'-ACTCCRACRCACRCACTACATCT-3'		
MT3-forward		5'-GACCTGCCCCTGCCCTTCTGGTGG-3'	69°C	219 bp
MT3-reverse		5'-GCTCCACACGGAGGGGTGCCTTCT-3'		
ACTB-forward		5'-TTGCCGACAGGATGCAGAAG-3'	59°C	101 bp
ACTB-reverse		5'-CTTTCCAAGCGGAGCCATGTCTGG-3'		
PBGD-forward		5'-CTTTCCAAGCGGAGCCATGTCTGG-3'	59°C	377 bp
PBGD-reverse		5'-CATGAGGGTTTTCCCGCTTGCAGA-3'		

^a ^Nucleotide position relative to the transcription start site.

### Analysis of *MT3 *expression by quantitative real-time RT-PCR

Cell line RNA was isolated using the RNeasy kit (Qiagen) with an on-column DNase I digestion. Tissue RNA was isolated using Trizol (Invitrogen, Mount Waverly, VIC, Australia) and treated with DNase I (Ambion). The cDNA was synthesised from 2 μg of RNA using an M-MLV kit (Invitrogen). Quantitative real-time RT-PCR was performed on a RotorGene 2000 PCR thermocycler (Corbett Research, Sydney, NSW, Australia). Triplicate reactions were done using the appropriate PCR primers (Table 4) and the Quantitect Sybr-Green PCR mix (Qiagen). The PCR products were electrophoresed on 1.5 % agarose gels and stained with ethidium bromide. The expression of *MT3 *was normalized to that of either *ACTB *or *PBGD *[25].

### Data analysis

Statistical analysis of expression of *MT3 *before and after aza-dC treatment for each cell line was performed using unpaired T-tests. Comparisons of the levels of *MT3 *between cell lines with or without aza-dC treatment were performed using ANOVA with Tukey-Kramer multiple comparisons test. The comparison of the volume of methylated and unmethylated tumours was performed using a Kruskal-Wallis Test. The comparison of patients with or without positive lymph nodes and methylation status was performed using a Chi-squared test. The Mann-Whitney test was used to compare *MT3 *expression in methylated and unmethylated tumour. Comparisons of *MT3 *expression in tumour and margin were performed using Wilcoxon matched-pairs sign-rank tests. Survival data were analysed using Fisher's Exact Test. All statistics were performed using InStat version 3.0a (GraphPad Software, San Diego, CA).

## List of abbreviations

aza-dC, 5-aza-2'-deoxycytidine; COBRA, combined bisulphite restriction analysis; MT3, metallothionein 3; RT-PCR, reverse transcriptase-polymerase chain reaction; SCC, squamous cell carcinoma.

## Authors' contributions

ES isolated the DNA and RNA, performed the bisulphite modifications, COBRAs and RT-PCRs, designed the study, performed the statistical analysis and coordinated and drafted the manuscript. GCM aided with the RT-PCRs. ZQT and JFL collected the oesophagectomy material and clinical data. NJD performed the cell culture and edited the manuscript. AR confirmed the pathology. PD, DIW and GGJ supervised the work and edited the manuscript. All authors read and approved the final manuscript.
